# Complete chloroplast genome sequence of the mangrove species *Kandelia obovata* and comparative analyses with related species

**DOI:** 10.7717/peerj.7713

**Published:** 2019-09-20

**Authors:** Yong Yang, Ying Zhang, Yukai Chen, Juma Gul, Jingwen Zhang, Qiang Liu, Qing Chen

**Affiliations:** 1 Ministry of Education Key Laboratory for Ecology of Tropical Islands, College of Life Sciences, Hainan Normal University, Haikou, China; 2 Life Sciences and Technology School, Lingnan Normal University, Zhanjiang, China; 3 Bawangling National Nature Reserve, Changjiang, Hainan Province, China

**Keywords:** Chloroplast genome, *Kandelia obovata*, Mangrove species, Phylogeny comparative analysis

## Abstract

As one of the most cold and salt-tolerant mangrove species, *Kandelia obovata* is widely distributed in China. Here, we report the complete chloroplast genome sequence *K. obovata* (Rhizophoraceae) obtained via next-generation sequencing, compare the general features of the sampled plastomes of this species to those of other sequenced mangrove species, and perform a phylogenetic analysis based on the protein-coding genes of these plastomes. The complete chloroplast genome of *K. obovata* is 160,325 bp in size and has a 35.22% GC content. The genome has a typical circular quadripartite structure, with a pair of inverted repeat (IR) regions 26,670 bp in length separating a large single-copy (LSC) region (91,156 bp) and a small single-cope (SSC) region (15,829 bp). The chloroplast genome of *K. obovata* contains 128 unique genes, including 80 protein-coding genes, 38 tRNA genes, 8 rRNA genes and 2 pseudogenes (*ycf1* in the IRA region and *rpl22* in the IRB region). In addition, a simple sequence repeat (SSR) analysis found 108 SSR loci in the chloroplast genome of *K. obovata*, most of which are A/T rich. IR expansion and contraction regions were compared between *K. obovata* and five related species: two from Malpighiales and three mangrove species from different orders. The mVISTA results indicated that the genome structure, gene order and gene content are highly conserved among the analyzed species. The phylogenetic analysis using 54 common protein-coding genes from the chloroplast genome showed that the plant most closely related to *K. obovata* is *Ceriops tagal* of Rhizophoraceae. The results of this study provide useful molecular information about the evolution and molecular biology of these mangrove trees.

## Introduction

*Kandelia obovata* Sheue, Liu & Yong, 2003 is a viviparous mangrove species belonging to Rhizophoraceae in Malpighiales that inhabits the intertidal zones of tropical and subtropical coasts. It is distributed from northern Vietnam through southeast China to south Japan in East Asia (Sheue, Liu & Yong, 2003; Tomlinson, 1986). This species is naturally distributed in the Hainan, Guangdong (including Hong Kong and Macau), Guangxi, Fujian and Taiwan Provinces of China (Li & Lee, 1997). With strong cold-resistance and high salt tolerance, *K. obovata* is one of the northernmost mangrove species in China (Bin-Yuan et al., 2007). Previous studies have found this species to have the ability to accumulate heavy metals (Weng et al., 2012; Weng et al., 2014). Previous studies of the molecular biology of *K. obovata* have focused on geographical relationships, genetic diversity (Chen et al., 2010) and cold stress (Fei et al., 2015). However, there has been no report of the chloroplast genome of *K. obovata*, which may be important for illuminating the evolution of mangrove species.

With the development of DNA sequencing technologies, an increasing number of researchers have focused on chloroplast genome research. Since the first two complete chloroplast genomes were reported from liverwort (Ohyama et al., 1986) and tobacco (Shinozaki et al., 1986), approximately 2,300 plant chloroplast genomes have been made publicly available in the National Center for Biotechnology Information (NCBI) database (https://www.ncbi.nlm.nih.gov/genome/browse#!/organelles/). Chloroplasts are organelles that provide energy to the plant and play an important role in photosynthesis and many biosynthetic activities (Douglas, 1998; Keeling, 2004). The structure of the chloroplast genome in most plants is characterized by a typical circular quadripartite structure and double-stranded DNA molecule, including a pair of inverted repeats (IRs) separated by a large single-copy region (LSC) and a small single-copy region (SSC) (Jansen et al., 2005). Generally, the chloroplast genome ranges from 107–218 kb in length and includes 110–130 genes, which are mainly involved in photosynthesis, transcription and translation; the gene content and gene order of the genome are highly conserved among taxa (Asaf et al., 2016; Daniell et al., 2016; Sugiura, 1992).

Up until the present study, 117 full plastid genome sequences from order Malpighiales had been published in NCBI, none of which are from mangrove plants species. Sequences of the true mangrove *Lumnitzera littorea* (MG182696) in Combretaceae, *Sonneratia alba* (MH105772) in Lythraceae and semi-mangrove *Barringtonia racemosa* (NC035705) in Lecythidaceae have been reported, but these species belong to Myrtales or Ericales. Comparisons of the chloroplast genome among *K. obovata* and mangrove species from different orders that also experience high salt and anoxic stress will improve our understanding of the evolution of stress tolerance.

In this study, we sequenced and analyzed the complete chloroplast genome of *K. obovata* based on next-generation sequencing methods (Illumina, HiSeq X Ten, San Diego, California, USA), and deposited the annotated sequence into the NCBI database under accession number MH277332. Then, the complete chloroplast genomes of six related species from different orders were compared to explore the evolution of the chloroplast genome. Subsequently, a phylogenomic analysis was performed based on the 54 protein-coding genes of 22 chloroplast genomes. Our study will improve the understanding of the evolutionary relationships among these mangrove species.

## Materials & Methods

### Sampling and DNA sequencing

Samples were collected in Xinyin National Wetland Park, Danzhou, China (19°30′N, 109°30′). Voucher specimens were deposited in the herbarium of Hainan Normal University under accession number Yang Y-201803, and replicate specimens (XY2019061201) were sent to Traditional Chinese Medicine Herbarium of Hainan Province for conservation. Five fresh leaves were collected from five healthy trees of *K. obovata* and then stored on ice for the return to the laboratory. Total genomic DNA was extracted from mixed fresh leaf tissues using the CTAB method (Doyle & Doyle, 1987). The extracted total genomic DNA was dissolved in 50 µL of TE buffer. After quality and concentration were analyzed by agarose electrophoresis and spectrophotometry (Beijin Puxi T6, China), a final DNA concentration of >30 ng/mL was used for Illumina sequencing. Library preparation and sequencing were performed at TGS-Shenzhen, China. The genome was sequenced on an Illumina HiSeq X Ten platform (Illumina, San Diego, CA, United States) with 150 bp paired-end reads.

### Chloroplast genome assembly and annotation

To ensure accurate and reliable analyses, raw data were proofread and assembled with NOVOPlast2.7.1 (Dierckxsens, Mardulyn & Smits, 2016) and verified by SOAPdenovo2 (Luo et al., 2012). A partial *rbcL* gene sequence of *Ceriops tagal* (MH240830) in Rhizophoraceae (Chen et al., 2019) was used as seed, and the chloroplast genome sequence of *C. tagal* was used as the reference sequence. The chloroplast genome of *K. obovata* was annotated by the Dual Organellar GenoMe Annotator program (DOGMA; http://dogma.ccbb.utexas.edu/) (Wyman, Jansen & Boore, 2004). The initial annotations and putative start, stop, and intron positions were checked manually based on comparison with the chloroplast genomes of *Erythroxylum novogranatense* (NC030601), as Erythroxylaceae is sister to Rhizophoraceae (Setoguchi, Kosuge & Tobe, 1999). Additionally, tRNA genes were identified by the tRNAscan-SE 1.21 program (Schattner, Brooks & Lowe, 2005). Physical maps were drawn using the web tool Organellar Genome DRAW (OGDRAW) v1.2 (Lohse, Drechsel & Bock, 2007).

### Simple sequence repeat (SSR) analysis

Distributed throughout the genome, SSRs are repeat sequences with a typical length of 1–6 bp that are generally considered to have a higher mutation rate than neutral DNA regions. The distributions of SSRs in the chloroplast genome were predicted by using the microsatellite search tool MISA (Kurtz et al., 2001) with the following parameters: ≥10 for mononucleotide repeats, ≥5 for dinucleotide repeats, ≥4 for trinucleotide repeats, and ≥3 for tetranucleotide repeats, pentanucleotide repeats, and hexanucleotide repeats.

### Comparative Genome analysis

To investigate the sequence divergence of the chloroplast genome among the analyzed mangrove species, the whole chloroplast genome sequences of the mangrove species *K. obovata* and *C. tagal* of Rhizophoraceae, *L. littorea* of Combretaceae, and *S. alba* of Lythraceae; the semi-mangrove species *B. racemosa* of Lecythidaceae; and the land species *E. novogranatense* of Erythroxylaceae were analyzed using the mVISTA program in the Shuffle-LAGAN mode (Frazer et al., 2004). The *K. obovata* annotations were used as references. The differences in the chloroplast genome length, LSC length, SSC length, GC content, encoding gene types and gene numbers among these 6 species were analyzed. The LSC/IR/SSC boundaries among the species were determined by comparative analysis to explore the variation in these angiosperm chloroplast genomes.

### Phylogenetic analysis

To understand the phylogenetic position of Rhizophoraceae in Malpighiales, we selected the chloroplast genome sequences of 22 other species published in the NCBI Organelle Genome Resource database and used 54 chloroplast protein-coding genes *atpA*, *atpB*, *atpE*, *atpF*, *atpH*, *atpI*, *ccsA*, *clpP*, *matK*, *ndhA*, *ndhE*, *ndhG*, *ndhH*, *ndhI*, *ndhJ*, *ndhK*, *petA*, *petD*, *petG*, *petL*, *petN*, *psaA*, *psaB*, *psaC*, *psaI*, *psaJ*, *psbA*, *psbC*, *psbD*, *psbF*, *psbH*, *psbJ*, *psbL*, *psbM*, *psbN*, *psbT*, *rbcL*, *rpl14*, *rpl16*, *rpl33*, *rpl36*, *rpoA*, *rpoB*, *rpoC1*, *rps11*, *rps14*, *rps15*, *rps18*, *rps2*, *rps3*, *rps4*, *rps8*, *ycf3*, *ycf4*) to construct a phylogenetic tree; *Linum usitatissimum* (NC_036356) of Linaceae were used as the outgroup. After downloading the chloroplast genome sequences from the NCBI database, the common 54 protein-coding genes among the 22 complete chloroplast genomes were aligned by MUSCLE (Edgar, 2004). Phylogenetic trees were constructed using maximum likelihood (ML) and maximum parsimony (MP) methods with MEGA7 software (Kumar, Stecher & Tamura, 2016) (https://www.megasoftware.net/), and the GTR+I+G model was used for the ML analysis. One thousand bootstrap replicates were used to calculate the local bootstrap probability of each branch.

## Results

### Genome organization and gene features

**Figure 1:**
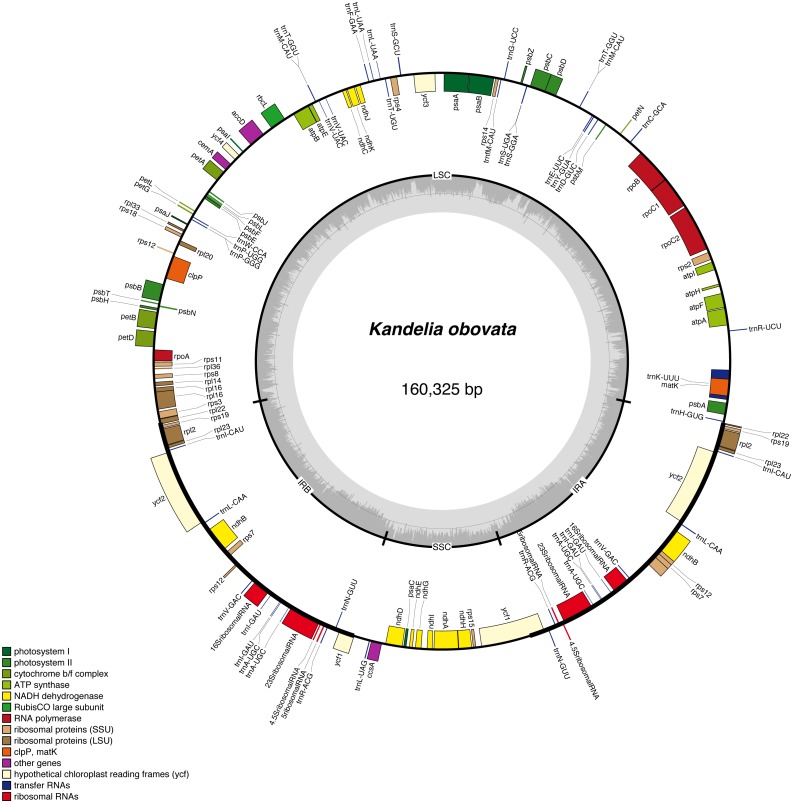
Gene map of the *K. obovata* chloroplast genome sequence. Genes shown outside the outer circle are transcribed clockwise, and genes shown inside the circle are transcribed counterclockwise. Genes belonging to different functional groups are color coded. The dashed area in the inner circle indicates the GC content of the chloroplast, and the light gray area corresponds to AT content of the chloroplast.

In total, we obtained 5,542 Mb short sequence data with a Q20 of 98.62% using the Illumina HiSeq X Ten platform. We obtained the same chloroplast genome sequence of *K. obovata* by the two methods (NOVOPlasty and SOAPdenovo2). The *K. obovata* chloroplast genome is a typical double-stranded circular DNA molecule with a quadripartite structure. The length is 160,325 bp (Fig. 1), with a pair of IR regions 26,670 bp in length that separate an LSC region of 91,156 bp and an SSC region of 15,829 bp. The GC content of the chloroplast genome is 35.23% (Table 1), and the GC contents of the LSC, SSC and IR regions are 32.34%, 29.05% and 41.99% respectively. As the rRNA genes *rrn23*, *rrn16*, *rrn5* and *rrn4.5* are located in the IR region, the IR region shows a higher GC content. The above-described content of the *K. obovata* chloroplast genome is similar to the contents of other chloroplast genomes in Malpighiales (de Santana Lopes et al., 2018; Li et al., 2017). However *K. obovata, C. tagal* and *E. novogranatense,* all of Malpighiales, have larger genome sizes and LSC sizes and lower GC contents than those mangrove species from different orders (Table 1).

**Table 1:** Summary of the complete chloroplast genome characteristics of 6 species.

**Species**	**Genome size(bp)**	**LSC size(bp)**	**SSC size(bp)**	**IR size(bp)**	**Number of genes**	**Protein coding genes**	**tRNA genes**	**rRNA genes**	**Number of genes duplicated**	**GC content (%)**	**GenBank No.**
*K. obovata*	160,325	91,156	15,829	26,670	130	84	38	8	19	35.23	MH277332
*C. tagal*	164,439	92,488	20,171	26,390	134	84	42	8	17	35.32	MH240830
*E. novogranatense*	163,937	91,383	18,138	27,208	131	85	38	8	18	35.89	NC030601
*L. littorea*	159,687	88,323	18,558	26,403	130	86	36	8	18	37.01	MG182696
*S. alba*	153,061	87,226	18,033	23,901	106	79	24	8	18	37.29	MH105772
*B. racemosa*	159,002	88,290	18,596	26,058	132	87	37	8	18	36.86	NC035705

The chloroplast genome contains a total of 128 unique genes, including 80 protein-coding genes, 38 tRNA genes, 8 rRNA genes and 2 pseudogenes. Among them, 19 of these genes occur in IRs, and contain 8 protein-coding genes (*rps19*, *rpl2*, *rpl23*, *ycf2*, *ndhB*, *rps7*, *rps12*, *ycf1*), 7 tRNA genes (*trnI-CAU*, *trnL-CAA*, *trnV-GAC*, *trnI-GAU*, *trnA-UGC*, *trnR-ACG*, *trnN-GUU*) and 4 rRNA genes (*rrn23*, *rrn16*, *rrn5*, *rrn4.5*). In total, 15 genes with introns were found. Thirteen of these genes contain one intron, and two of these genes (*clp3* and *ycf3*) contain two introns (Table 2). *Rps12* is a trans-spliced gene with a 5′ exon located in an LSC region and two 3′ exons located in IR regions, similar to most other plant chloroplast genomes (Asaf et al., 2016; Cheng et al., 2017; Raman & Park, 2016; Jiang et al., 2017). The start of the nucleotide sequence of a protein-coding gene usually begins with ATG. However, there are some exceptions in the *K. obovata* chloroplast genome in which the first nucleotide has changed from A to G or the second nucleotide has changed from T to C, such as *rps19* and *cemA*, which begin with GTG, and *ndhD* which begin with ACG. The *K. obovata* chloroplast genome with gene annotations was submitted to GenBank under the accession number MH277332.

**Table 2:** List of annotated genes in the chloroplast genome of *K. obovata*.

**Category**	**Group of genes**	**Name of genes**
Self-replication	Large subunit of ribosomal proteins	rpl2*2^b^^c^, *14,16*^b^, *20,22*^c^, 23*2^c^, *33,36*
	Small subunit of ribosomal proteins	rps2,3,4,7*2^c^, 8,11,12*2^a^^c^, 14,15,18,19*2^c^
	DNA dependent RNA polymerase	*rpo* A, B, C1^b^, C2
	rRNA genes	*rrn 4.5* *2^c^, *5* *2^c^, *16* *2^c^, *23* *2^c^
	tRNA genes	*trnA-UGC* *2^b^^c^, *trnC-GCA*, *trnD-GUC*, *trnE-UUC*, *trnF-GAA*, *trnfM-CAU*, *trnG-UCC*, *trnH-GUG*, trnI-CAU* 2^c^, *trnI-GAU* *2^b^^c^, * trnK-UUU*^b^, *trnL-CAA* *2^c^, *trnL-UAA*^b^, *trnL-UAG*, *trnM-CAU* *2, *trnN-GUU* *2^c^, *trnP-GGG*, *trnP-UGG*, *trnR-ACG* *2^c^, *trnR-UCU*, *trnS-GCU*, *trnS-GGA*, *trnS-UGA*, *trnT-GGU*, *trnT-UGU*,*trnT-UGU*, *trnV-GAC* *2^c^, *trnV-UAC*^b^, *trnW-CCA*, *trnY-GUA*
Photosynthesis	Subunits of Photosystem I	*psaA*, *B*, *C*, *I*, *J*
	Subunits of Photosystem II	*psbA*, *B*, *C*, *D*, *E*, *F*, *H*, *J*, *L*, *M*, *N*, *T*, *Z*
	Subunits of NADH dehydrogenes	*ndhA*^b^, *B* *2^b^^c^, *C*, *D*, *E*, *G*, *H*, *I*, *J*, *K*
	Cytovchrome b6/f complex	*petA*, *B*^b^, *D*^b^, *G*, *L*, *N*
	ATP synthase	*atpA*, *B*, *E*, *F*^b^, *H*, *I*
	Rubisco	*rbcL*
Other genes	Maturase	*matK*
	Subunit Acrtyl-CoA-Carboxylate	*accD*
	Envelop membrane protein	*cemA*
	Proteaese	*clpP*^a^
	c-type cytochrome synthesis gene	*ccsA*
Unknown	Conserved Open reading frames	*ycf1*^b^, *2* *2^c^, *3*^a^, *4*
	pseudogene	*ycf1*, *rpl22*

**Notes.**

a Gene with two introns.

b Gene with one intron.

c Genes located in the inverted repeats.

### Simple sequence repeats (SSR) analysis

**Figure 2:**
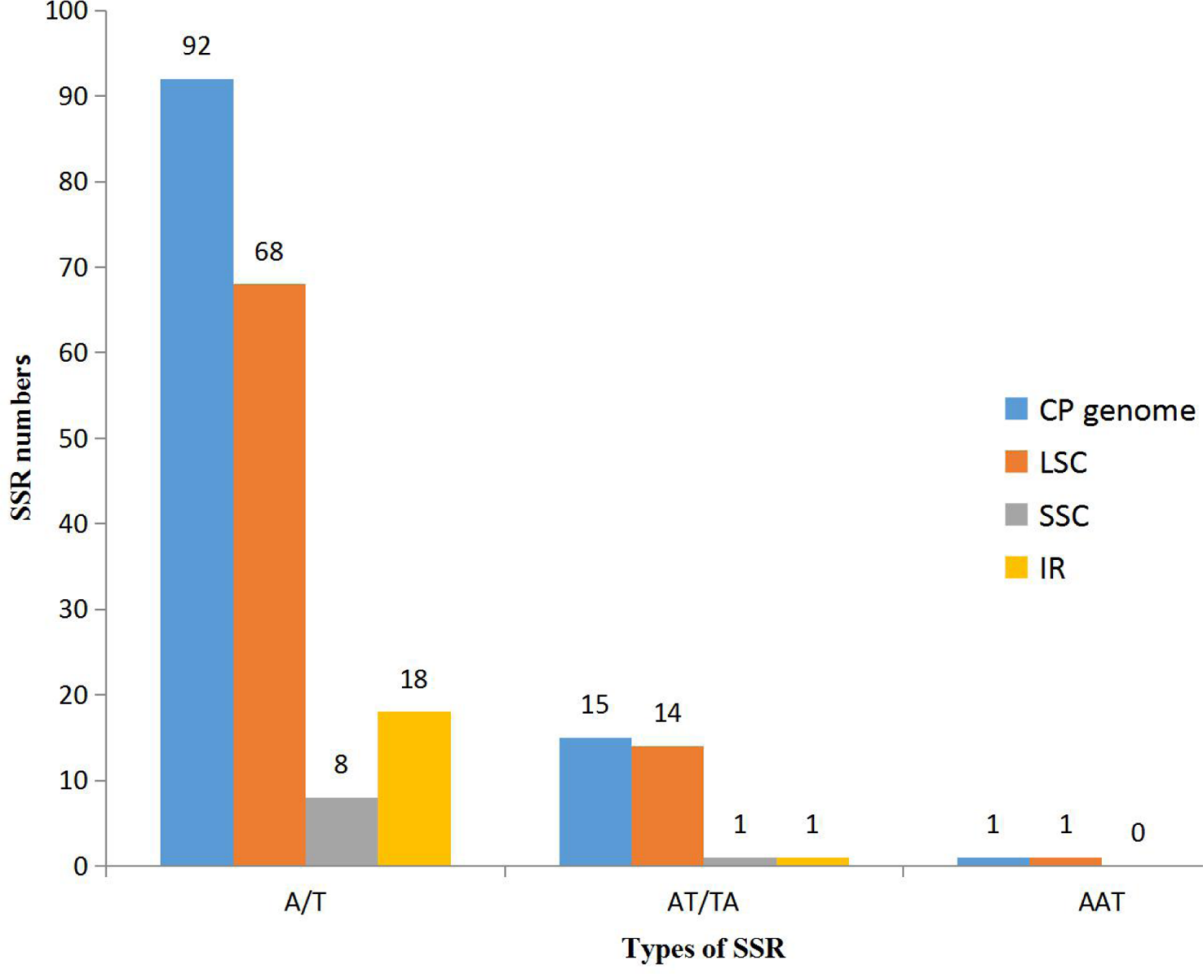
The distribution of SSRs in three regions: LSC, SSC and IR regions.

With the development of next generation sequencing (NGS) technologies, SSR development has become quicker, more efficient and cheaper than before, even in species for which background genetic information is lacking (Davey et al., 2011; Zalapa et al., 2012). In our research, using the microsatellite identification tool MISA, we identified 108 SSR loci in the *K. obovata* complete chloroplast genome sequence, including 92 mononucleotide SSR loci (A/T), 15 dinucleotide SSR loci (AT/TA), and 1 trinucleotide SSR loci (AAT) (Table 3). A total of 103 of the 108 SSRs are located in intergenic regions, and 5 SSRs are located in gene-coding regions. Furthermore, 83, 6 and 19 SSRs, were discovered within the LSC, SSC and IR regions, respectively (Fig. 2).

**Table 3:** Summary of the number of nucleotide repeat units in chloroplast gene of *K. obovata*.

**Repeat motif**			**Number of repeats**								
		**5**	**6**	**7**	**8**	**9**	**10**	**11**	**12**	**≥13**	**Total**
Mono-nucleotide	A/T	–	–	–	–	–	42	19	20	11	92
Di-nucleotide	AT/TA	–	10	3	1	1	–	–	–	–	15
Tri-nucleotide	AAT/TTA	1	–	–	–	–	–	–	–	–	1
Total		1	10	10	9	10	52	30	32	11	108
Ratio (%)		0.93%	9.26%	9.26%	8.33%	9.26%	48.15%	27.78%	29.63%	10.19%	100.0%

**Notes.**

a ‘–’ represents no repeat unit.

### Comparative analysis of the chloroplast genome sequences of six species

**Figure 3:**
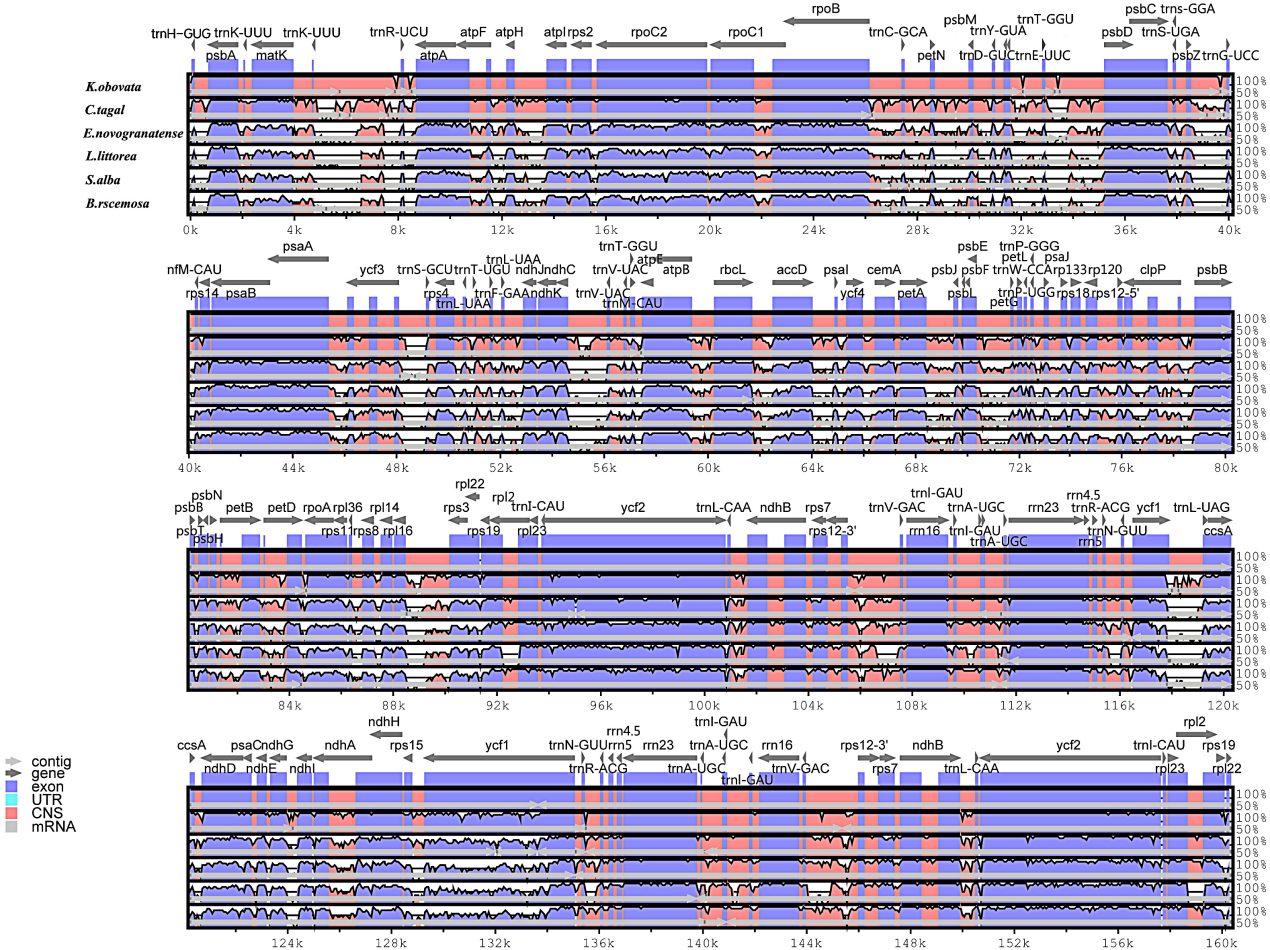
Visualization alignment of the chloroplast genome sequence of six species. The identity percentages are shown in the *y*-axis and range from 50% to 100%, while the horizontal axis shows the position within the chloroplast genome. Each arrow indicates the annotated genes and direction of their transcription in the reference genome. Genome regions, i.e., exons, untranslated regions (UTRs), conserved noncoding sequences (CNS) and mRNA, are color coded.

In this study, the chloroplast genomes of several mangrove species and one land species were analyzed by the mVISTA program. Considerable similarities in genome composition and size were identified among the species (Fig. 3). The coding regions of the two mangrove species in Rhizophoraceae were almost identical, whereas the non-coding regions are more variable. The mangrove species in Malpighiales shows a closer relationship with the land plant *E. novogranatense*, which belongs to the same order, than to the mangroves species from different orders.

### IR contraction and expansion

**Figure 4:**
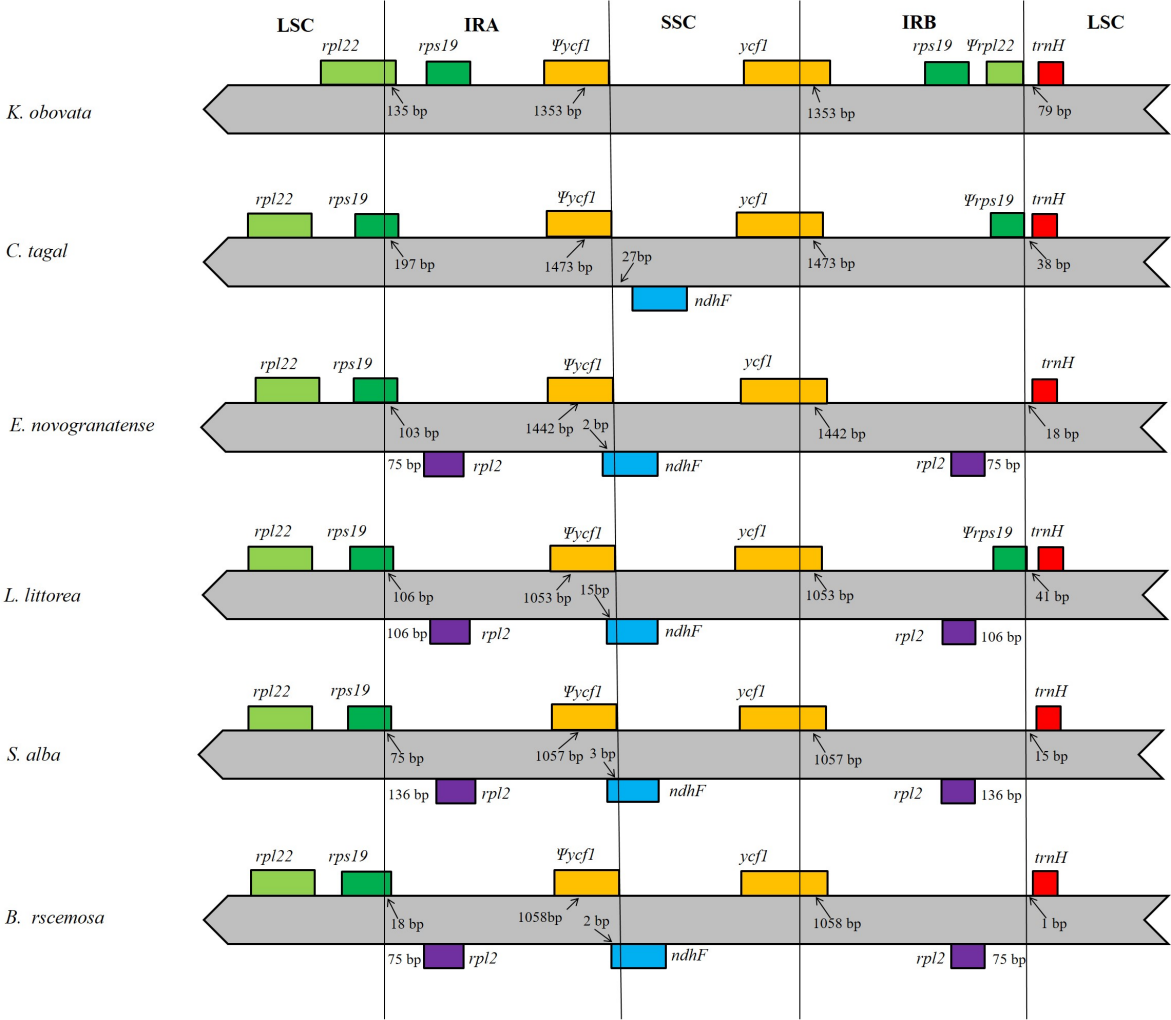
Comparison of the boundaries of the LSC, SSC and IR regions of the five chloroplast genomes. The numbers above the gene features indicate the distance between the ends of genes and the border sites. Those features are not to scale.

In this study, we aligned the positions of the LSC, IRA, SSC and IRB borders and the adjacent genes among members of mangrove species and found that the studied locations are generally similar to those of all previously reported chloroplast genomes (Jo et al., 2016; Wei et al., 2017; Zhou et al., 2017; Yu et al., 2018). However, in the analyzed species, two copies of the *K. obovata rpl22* gene are located in the boundaries of the LSC/IRA junction and IRB region; as the *rpl22* gene in the IRB region has no open reading frame that encodes a functional protein, we regarded this gene as a pseudogene. However, in the other species, *rpl22* is only located in the LSC region, and there is no *rpl22* in the IRB region. The IR extended into the *ycf1* genes, creating long *ycf1* pseudogenes with variable lengths. The length of the *ycf* 1 pseudogene is in the IRA region is 1,353 bp, whereas the *ycf1* in the SSC/IRB junction contains intact open reading frames (ORFs). There are two copies of *rps19* in the IR region of the *K. obovata* chloroplast genome, but in the other species, there is only one copy, located in the junction of the LSC/IRA region. In all the analyzed genomes, the *trnH* gene is the first gene in the LSC region, although its distance from the IRB/LSC junction ranges from 1 bp to 79 bp. Comparisons among the species revealed that the *K. obovata* chloroplast genome has lost the *ndhF* gene (Fig. 4).

### Phylogenetic analysis

**Figure 5:**
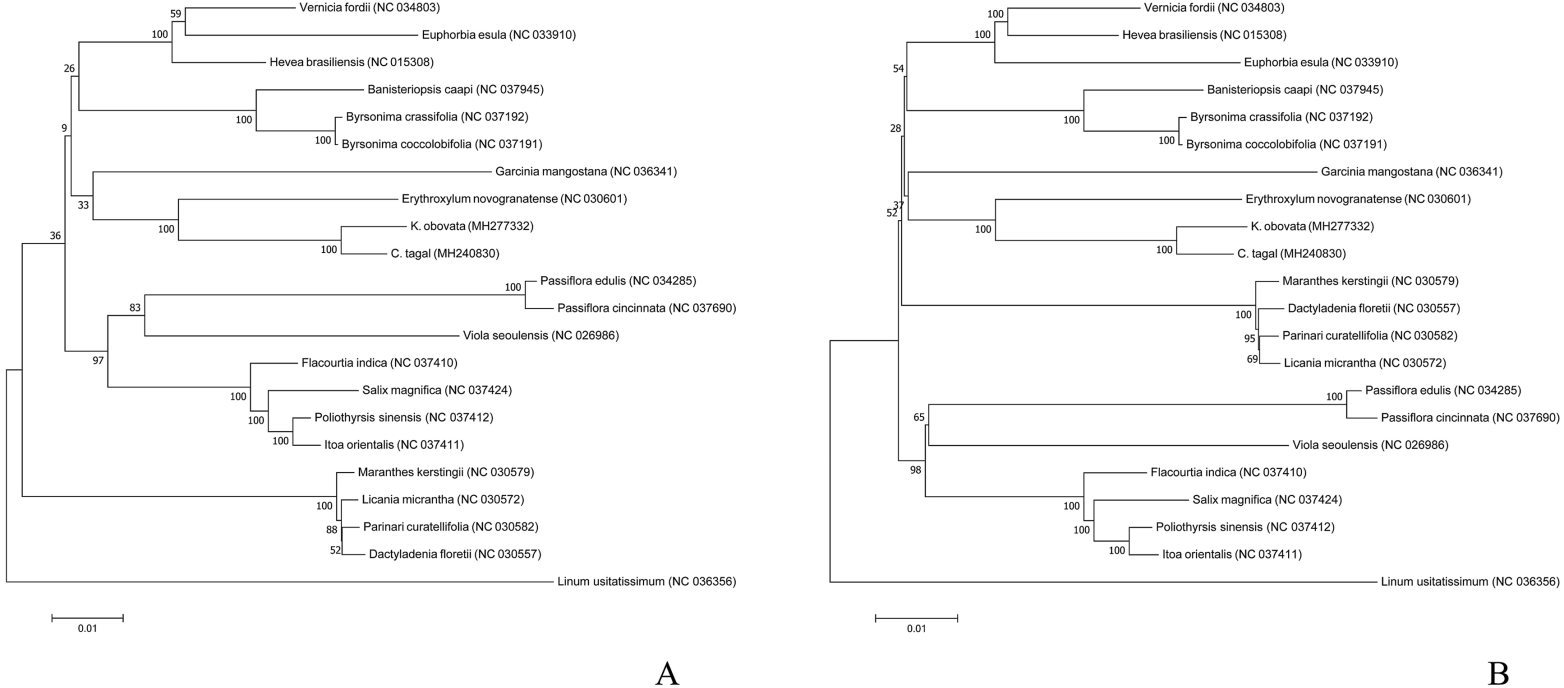
Phylogenetic trees inferred via (A) ML and (B) MP based on the coding sequences of 54 protein-coding genes from 22 species and using *Linum usitatissimum* as the outgroup.

To analyze the phylogenetic position of *K. obovata* in Malpighiales, we used the common 54 protein-coding genes among the 22 complete chloroplast genome sequences to infer phylogenetic relationships. The phylogenetic trees generated by the ML and MP methods have similar topologies (Fig. 5). Both show that *K. obovata* is most closely related to *C. tagal,* with 100% bootstrap supports; both of these taxa belong to *Rhizophoraceae.* These two species are more closely related to the land species *E. novogranatense* (Erythroxylaceae) than to the other species, suggesting that Erythroxylaceae is sister to Rhizophoraceae. Our study will provide valuable genetic information for genome-scale phylogenetic studies in mangrove plants.

## Discussion

With the development of NGS, chloroplast genome sequences can be obtained efficiently and economically. In the present study, we obtained the complete sequence of the *K. obovata* chloroplast genome (160,325 bp), which was fully characterized and compared to the chloroplast genomes of species from different orders. The *K. obovata* chloroplast genome includes 128 unique genes encoding 80 proteins, 8 rRNAs, 38 tRNAs and two 2 pseudogenes (*ycf1* in the IRA region and *rpl22* in the IRB region). *rps19* and *cemA* begin with GTG, and *ndhD* begin with ACG; these changes are most likely made at the RNA stage. Similar exceptions have been found in the chloroplast genomes of other plants, such as *Betula platyphylla* (Wang et al., 2018), *Panax ginseng* (Zhao et al., 2015) and *Phoenix dactylifera* L. (Yang et al., 2010). The obtained chloroplast genome of *K. obovata* has a typical quadripartite structure, and its gene content, gene order and GC content are similar to those of most other species from different orders. Although IRs are more conservative than the LSC and SSC regions in chloroplast DNA, the expansion and contraction of the border between SC and IR regions are common evolutionary events and produce size variation in chloroplast genomes (Raubeson et al., 2007; Wang et al., 2008). Relative to the IR regions of other mangrove species, those of *K. obovata* contain an additional gene (*rps19*). Furthermore, *K. obovata* shows a lower GC content than the other mangrove species *L. littorea*, *S. alba* and *B. racemosa* of Myrtales.

Simple sequence repeats (SSRs) are significant repetitive elements of the entire genome and play important roles in genome recombination and rearrangement. The SSRs in chloroplast genomes are usually distributed in intergenic regions (Zhao et al., 2015). In the SSR analysis, 108 SSR loci were found, and most SSRs were located in the intergenic region. The SSRs identified in the chloroplast genome of *K. obovata* can be used to analyze polymorphisms at the intraspecific level. They can also be used to develop lineage-specific markers for future evolutionary and genetic diversity studies. The mVISTA results showed that the sequence of the chloroplast genome is highly conserved among the six species. In addition, they showed that the sequence and content of IR regions are more conserved than are those of the LSC and SSC regions among the studied species, possibly because of the rRNA in IR regions. The results showed that the genes of the chloroplast genome are largely identical between *K. obovata* and *C. tagal*, whereas the intergenic regions are more variable. Thus, we propose that the intergenic regions could potentially be used as molecular markers (such as *trnL-trnF*, *petA-psbJ* and *ndhC-trnV*) for evolutionary and genetic diversity studies of these two species and other species of Rhizophoraceae.

In this study, the phylogenetic position of *K. obovata* in the Malpighiales was inferred by analyzing the complete chloroplast genome and 54 genes shared among 22 species. The results suggest that *K. obovata* is most closely related to *C. tagal* of Rhizophoraceae and *E. novogranatense* of Erythroxylaceae despite their contrasting living habitats. The bootstrap values were high for species reconstructed in the same family but were low among different families, suggesting that these protein-coding genes may be conserved within families but vary extensively among families. In addition, the mVISTA results showed that *E. novogranatense* is more closely related to the species in Rhizophoraceae than to other mangrove species despite having very different living conditions. Compared with the land species, the mangrove species have evolved features such as salt glands and pneumathodium as adaptations to their high salt, anoxic environments. Although *K. obovata* is a typical mangrove plant occurring in the coastal intertidal zone, the characteristics of this plant are quite similar to those of land species of angiosperm (Asaf et al., 2017; Bruneau, Doyle & Palmer, 1990; Sugiura, 1992).

We found no character in the chloroplast genome that distinguished the land species from the mangrove species, even though the species in Rhizophoraceae are generally considered mangrove species living in intertidal environments. Therefore, we infer that genetic variation within the chloroplast genome did not contribute to the adaptation of different genera to divergent habitats. Rhizophoraceae includes some land species, such as those belonging to the genera *Carallia* Roxb. and *Pellacalyx* Korth. The extent of genetic variation of plastomes among mangrove and non-mangrove species does not correspond to habitat divergence among these taxa. Further studies of this topic are warranted. Our study on the *K. obovata* chloroplast genome provides information on mangrove plant species in coastal intertidal zones. Moreover, the chloroplast genomic data provided in this study will be valuable for future phylogenetic studies and other studies of mangrove species.

## Conclusion

We successfully assembled, annotated and analyzed the complete chloroplast sequence of *K. obovata*, a mangrove species. The chloroplast genome was found to be conserved among several species, with that of *K. obovata* being very similar to both other mangrove species and land species. We identified 108 SSR loci in the chloroplast, which can be used for the development of lineage-specific markers The LSC/IRB/SSC/IRA boundary regions of the chloroplast genome were compared among four mangrove species, and the results revealed that the *K. obovata* chloroplast genome has lost the *ndhF* gene. The phylogenetic analyses showed that *K. obovata* is most closely related to *C. tagal* among the studied taxa. The molecular data in this study represent a valuable resource for the study of evolution in mangrove species.

##  Supplemental Information

10.7717/peerj.7713/supp-1 Supplemental Information 1 Kandelia obovata sequence data

## Additional Information and Declarations

### Data Availability

The following information was supplied regarding data availability: The raw data are available in File S1. The *Kandelia obovata* chloroplast genome with gene annotations was submitted to GenBank under the accession number MH277332.

## References

[ref-1] Asaf S, Khan AL, Khan AR, Waqas M, Kang SM, Khan MA, Lee SM, Lee IJ (2016). Complete chloroplast genome of *Nicotiana otophoraand* and its comparison with related species. Frontiers in Plant Science.

[ref-2] Asaf S, Waqas M, Khan AL, Khan MA, Kang SM, Imran QM, Shahzad R, Bilal S, Yun BW, Lee IJ (2017). The complete chloroplast genome of wild rice (*Oryza minuta*) and its comparison to related species. Frontiers in Plant Science.

[ref-3] Bin-Yuan HE, Fan HQ, Mao W, Lai TH (2007). Species diversity in mangrove wetlands of China and its causation analyses. Acta Ecologica Sinica.

[ref-4] Bruneau A, Doyle JJ, Palmer JD (1990). A chloroplast DNA inversion as a subtribal character in the phaseoleae (Leguminosae). Systematic Botany.

[ref-5] Chen SB, Ding WY, Qiu JB, Wang GY, Zhou ZM, Chen JF, Ai WM, Wang CY, Xie QL (2010). The genetic diversity of the mangrove *Kandelia obovata* in China revealed by ISSR analysis. Pakistan Journal of Botany.

[ref-6] Chen YK, Yang Y, Li JW, Jin YH, Liu Q, Zhang Y (2019). The complete chloroplast genome sequence of a medicinal mangrove tree *Ceriops tagal* and its phylogenetic analysis. Mitochondrial DNA Part B.

[ref-7] Cheng H, Li J, Zhang H, Cai B, Gao Z, Qiao Y, Mi L (2017). The complete chloroplast genome sequence of strawberry (*Fragaria*×*ananassa* duch.) and comparison with related species of *Rosaceae*. Peerj.

[ref-8] Daniell H, Lin CS, Ming Y, Chang WJ (2016). Chloroplast genomes: diversity, evolution and applications in genetic engineering. Genome Biology.

[ref-9] Davey JW, Hohenlohe PA, Etter PD, Boone JQ, Catchen JM, Blaxter ML (2011). Genome-wide genetic marker discovery and genotyping using next-generation sequencing. Nature Reviews Genetic.

[ref-10] de Santana Lopes A, Pacheco TG, Santos KGD, Vieira LDN, Guerra MP, Nodari RO, de Souza EM, de Oliveira Pedrosa F, Rogalski M (2018). The *Linum usitatissimum* L. plastome reveals atypical structural evolution, new editing sites, and the phylogenetic position of Linaceae within Malpighiales. Plant Cell Reports.

[ref-11] Dierckxsens N, Mardulyn P, Smits G (2016). NOVOPlasty: *de novo* assembly of organelle genomes from whole genome data. Nucleic Acids Research.

[ref-12] Douglas SE (1998). Plastid evolution: origins, diversity, trends. Current Opinion in Genetics & Devolopment.

[ref-13] Doyle JJ, Doyle JL (1987). A rapid DNA isolation procedure for small quantities of fresh leaf tissue. Phytochemical Bulletin.

[ref-14] Edgar RC (2004). MUSCLE: multiple sequence alignment with high accuracy and high throughput. Nucleic Acids Research.

[ref-15] Fei J, Wang YS, Jiang ZY, Cheng H, Zhang JD (2015). Identification of cold tolerance genes from leaves of mangrove plant *Kandelia obovata* by suppression subtractive hybridization. Ecotoxicology.

[ref-16] Frazer KA, Pachter L, Poliakov A, Rubin EM, Dubchak I (2004). VISTA: computational tools for comparative genomics. Nucleic Acids Research.

[ref-22] Jansen RK, Raubeson LA, Boore JL, Depamphilis CW, Chumley TW, Haberle RC, Wyman SK, Alverson AJ, Peery R, Herman SJ, Fourcade HM, Kuehl JV, McNeal JR, Leebens-Mack J, Cui L (2005). Methods for obtaining and analyzing whole chloroplast genome sequences. Methods in Enzymology.

[ref-23] Jiang D, Zhao Z, Zhang T, Zhong W, Liu C, Yuan Q, Huang L (2017). The chloroplast genome sequence of *Scutellaria baicalensis* provides insight into intraspecific and interspecific chloroplast genome diversity in *Scutellaria*. Gene.

[ref-24] Jo S, Kim HW, Kim YK, Cheon SH, Kim KJ (2016). Complete plastome sequence of *Psidium guajava* L. (Myrtaceae). Mitochondrial DNA Part B.

[ref-25] Keeling PJ (2004). Diversity and evolutionary history of plastids and their hosts. American Journal of Botany.

[ref-26] Kumar S, Stecher G, Tamura K (2016). MEGA7: molecular evolutionary genetics analysis version 7.0 for bigger datasets. Molecular Biology & Evolution.

[ref-27] Kurtz S, Choudhuri JV, Ohlebusch E, Schleiermacher C, Stoye J, Giegerich R (2001). REPuter: the manifold applications of repeat analysis on a genomic scale. Nucleic Acids Research.

[ref-28] Li MS, Lee SY (1997). Mangroves of China: a brief review. Forest Ecology & Management.

[ref-29] Li Z, Long HX, Zhang L, Liu ZM, Cao HP, Shi MW, Tan XF (2017). The complete chloroplast genome sequence of tung tree (*Vernicia fordii*): organization and phylogenetic relationships with other angiosperms. Scientific Reports.

[ref-31] Lohse M, Drechsel O, Bock R (2007). OrganellarGenomeDRAW (OGDRAW): a tool for the easy generation of high-quality custom graphical maps of plastid and mitochondrial genomes. Current Genetics.

[ref-32] Luo R, Liu B, Xie Y, Li Z, Huang W, Yuan J, He G, Chen Y, Pan Q, Liu Y, Tang J, Wu G, Zhang H, Shi Y, Liu Y, Yu C, Wang B, Lu Y, Han C, Cheung DW, Yiu SM, Peng S, Xiaoqian Z, Liu G, Liao X, Li Y, Yang H, Wang J, Lam TW, Wang J (2012). SOAPdenovo2: an empirically improved memory-efficient short-read de novo assembler. GigaScience.

[ref-34] Ohyama K, Fukuzawa H, Kohchi T, Shirai H, Sano T, Sano S, Umesono K, Shiki Y, Takeuchi M, Chang Z, Aota SI, Inokuchi H, Ozeki H (1986). Chloroplast gene organization deduced from complete sequence of liverwort *Marchantia polymorpha* chloroplast DNA. Nature.

[ref-18] Raman G, Park SJ (2016). The complete chloroplast genome sequence of ampelopsis: gene organization, comparative analysis, and phylogenetic relationships to other angiosperms. Frontiers in Plant Science.

[ref-35] Raubeson LA, Peery R, Chumley TW, Dziubek C, Fourcade HM, Boore JL, Jansen RK (2007). Comparative chloroplast genomics: analyses including new sequences from the angiosperms *Nuphar advena* and *Ranunculus macranthus*. BMC Genomics.

[ref-36] Schattner P, Brooks AN, Lowe TM (2005). The tRNAscan-SE, snoscan and snoGPS web servers for the detection of tRNAs and snoRNAs. Nucleic Acids Research.

[ref-19] Setoguchi H, Kosuge K, Tobe H (1999). Molecular phylogeny of rhizophoraceae based on *rbcL* gene sequences. Journal of Plant Research.

[ref-37] Sheue CR, Liu HY, Yong JWH (2003). *Kandelia obovata* (Rhizophoraceae), a new mangrove species from Eastern Asia. Taxon.

[ref-38] Shinozaki K, Ohme M, Tanaka M, Wakasugi T, Hayashida N, Matsubayashi T, Zaita N, Chunwongse J, Obokata J, Yamaguchi-Shinozaki K, Ohto C, Torazawa K, Meng BY, Sugita M, Deno H, Kamogashira T, Yamada K, Kusuda J, Takaiwa F, Kato A, Tohdoh N, Shimada H, Sugiura M (1986). The complete nucleotide sequence of the tobacco chloroplast genome: its gene organization and expression. Plant Molecular Biology Reporter.

[ref-39] Sugiura M (1992). The chloroplast genome. Plant Molecular Biology.

[ref-40] Tomlinson PB (1986). The botany of mangroves.

[ref-41] Wang RJ, Cheng CL, Chang CC, Wu CL, Su TM, Chaw SM (2008). Dynamics and evolution of the inverted repeat-large single copy junctions in the chloroplast genomes of monocots. BMC Evolutionary Biology.

[ref-42] Wang S, Yang C, Zhao X, Chen S, Qu G (2018). Complete chloroplast genome sequence of *Betula platyphylla*: gene organization, RNA editing, and comparative and phylogenetic analyses. BMC Genomics.

[ref-43] Wei LN, Cai YC, Wu W, Zhou RC (2017). The complete chloroplast genome sequence of *Melastoma candidum* (Melastomataceae). Mitochondrial DNA Part B.

[ref-44] Weng B, Huang Y, Liu J, Lu H, Yan C (2014). Alleviated toxicity of cadmium by the rhizosphere of *Kandelia obovata* (S. L.) Yong. Bulletin of Environmental Contamination & Toxicology.

[ref-45] Weng B, Xie X, Weiss DJ, Liu J, Lu H, Yan C (2012). *Kandelia obovata* (S. L.) Yong tolerance mechanisms to Cadmium: subcellular distribution, chemical forms and thiol pools. Marine Pollution Bulletin.

[ref-46] Wyman SK, Jansen RK, Boore JL (2004). Automatic annotation of organellar genomes with DOGMA. Bioinformatics.

[ref-47] Yang M, Zhang X, Liu G, Yin Y, Chen K, Yun Q, Chen K, Yun Q, Zhao D, AI-Messallem I, Yu J (2010). The complete chloroplast genome sequence of date palm (*Phoenix dactylifera* L.). PLOS ONE.

[ref-48] Yu TH, Hinsinger DD, Strijk JS, Wee AKS (2018). The first complete chloroplast genome of a major mangrove species *Sonneratia alba* Sm. and its implications on conservation efforts. Mitochondrial DNA Part B.

[ref-49] Zalapa JE, Cuevas H, Zhu H, Steffan S, Senalik D, Zeldin E, Mccown B, Harbut R, Simon P (2012). Using next-generation sequencing approaches to isolate simple sequence repeat (SSR) loci in the plant sciences. American Journal of Botany.

[ref-50] Zhao Y, Yin J, Guo H, Zhang Y, Xiao W, Sun C, Wu J, Qu X, Yu J, Wang X, Xiao J (2015). The complete chloroplast genome provides insight into the evolution and polymorphism of *Panax ginseng*. Frontiers in Plant Science.

[ref-51] Zhou QJ, Chen YM, Wu W, Zhou RC, Zhang Y (2017). The complete chloroplast genome sequence of an endangered mangrove tree *Lumnitzera littorea* (Combretaceae). Conservation Genetics Resources.

